# Cancer core modules identification through genomic and transcriptomic changes correlation detection at network level

**DOI:** 10.1186/1752-0509-6-64

**Published:** 2012-06-12

**Authors:** Wenting Li, Rui Wang, Linfu Bai, Zhangming Yan, Zhirong Sun

**Affiliations:** 1 MOE Key Laboratory of Bioinformatics, State Key Laboratory of Biomembrane and Membrane Biotechnology, Institute of Bioinformatics and Systems Biology, School of Life Sciences, Tsinghua University, Beijing, China; 2 Computational Biology and Bioinformatics Program, Institute for Genome Science and Policy, Duke University Medical Center, Durham, NC, USA

**Keywords:** Cancer core modules, Genotype-phenotype correlation, Network analysis

## Abstract

**Background:**

Identification of driver mutations among numerous genomic alternations remains a critical challenge to the elucidation of the underlying mechanisms of cancer. Because driver mutations by definition are associated with a greater number of cancer phenotypes compared to other mutations, we hypothesized that driver mutations could more easily be identified once the genotype-phenotype correlations are detected across tumor samples.

**Results:**

In this study, we describe a novel network analysis to identify the driver mutation through integrating both cancer genomes and transcriptomes. Our method successfully identified a significant genotype-phenotype change correlation in all six solid tumor types and revealed core modules that contain both significantly enriched somatic mutations and aberrant expression changes specific to tumor development. Moreover, we found that the majority of these core modules contained well known cancer driver mutations, and that their mutated genes tended to occur at hub genes with central regulatory roles. In these mutated genes, the majority were cancer-type specific and exhibited a closer relationship within the same cancer type rather than across cancer types. The remaining mutated genes that exist in multiple cancer types led to two cancer type clusters, one cluster consisted of three neural derived or related cancer types, and the other cluster consisted of two adenoma cancer types.

**Conclusions:**

Our approach can successfully identify the candidate drivers from the core modules. Comprehensive network analysis on the core modules potentially provides critical insights into convergent cancer development in different organs.

## Background

Cancer occurs when cells grow out of control due to genetic mutations [1]. It is not a single disease, but exhibits a wide spectrum of phenotypic variations involving numerous critical genes and pathways, e.g. TGF-β, NK-κB, TNF-α that may play multiple and even opposite roles [2,3]. Accordingly, a wide range of genetic mutations is involved, and the same mutations may exhibit a different impact. Further elucidation of the functional link between the genetic mutations and phenotypic changes in cancer development is of central importance, but remains a challenge [4]. Moreover, these genetic mutations disrupt the DNA repair pathways, resulting in many associated non-functional mutations [5].

Thus, this poses a big challenge to the central goal in cancer research to identify functional and critical mutations. Historically, identification has been approached starting with frequent single genes to pathways, followed by the identification of multiple gene modules. A previous study of 11 breast cancer samples and 11 colon cancer samples [6] focused on individual frequent mutated genes, and identified 189 candidate cancer genes (CAN genes), but failed to identify critical infrequent mutated genes. Cancer heterogeneity or insufficient tumor sample size contributed to this and complicated the efforts to distinguish the additional core mutations from the most infrequent background mutations. At the same time, both the functional “driver” mutations and the associated “passenger” mutations occur rarely; therefore, it is nearly impossible to distinguish them solely based on the frequency of individual genes [7,8]. Recent reports have focused on mining the over-represented mutations in co-expressed gene sets or modules [9]. In one recent report, the authors hypothesized that mutations cause cancer by disrupting certain functional modules [10], i.e., sets of genes involved in the same biological functions, via different combinations of infrequently mutated genes in the same module, as functionally linked genes tend to share similar mutational phenotypes [11-15].

In this study, our principle hypothesis is that cellular networks are comprised of many functionally related modules, and that critical genomic alterations may not necessarily change expression themselves, but may affect downstream functional modules expression levels and perturb normal functionality, leading to significant phenotypic changes.

Theoretically, from the network perspective, critical cancer gene disruption will cause a significant change in expression within its neighborhood and significant phenotypic changes. Driver mutations are defined as having been positively selected and contributing to cancer development; but conversely passenger mutations do not confer cancer development advantage [7]. One crucial form of phenotypic change during cancer development that has been extensively studied is change in gene expression [16]. And it has been demonstrated that the pathways or modules enriched for CAN genes are accompanied by significant expression changes [17]. Furthermore, some reports have identified candidate disease genes by searching through the significantly disrupted modules, which consist of their neighborhoods, using only gene expression information [18,19]. As a result, these top ranked significantly differentially expressed genes or modules represent the cancer phenotype changes, whereas upstream genomic alterations aid in locating the changing source.

Thus, based on this hypothesis, in order to discover the drivers we proposed a novel network-based approach to identify the core modules exhibiting both mutation enrichment and significant expression changes. The feasibility of this hypothesis was further assessed and genotype-phenotype correlations were detected at the network modular level. We applied this approach on six cancer types (ten datasets) to identify the core modules and demonstrate the utility of the method to correctly identify the known drivers. Furthermore, comprehensive network analyses were performed to further mine the identified driver network properties: mutations in the core modules tended to mutate the hub genes that exhibited central regulatory roles. Many of them were cancer type specific and relatively functionally isolated from those found in other cancer types. Only a few, reflecting the phylogenies of the six cancer types, played a general role in multiple cancer types.

## Results

### Identification of significant expression changed functional modules

**Figure 1:**
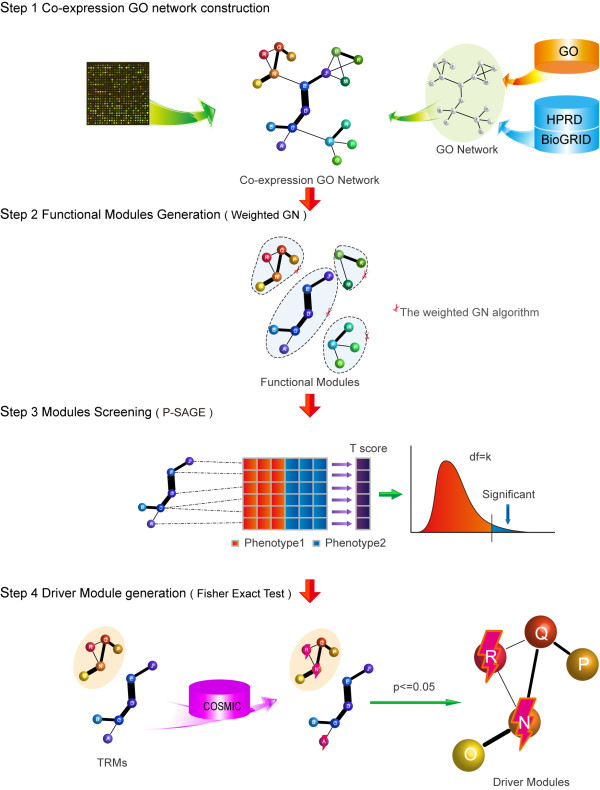
**Scheme of TRMs and core modules identification.** The first step is to construct the co-expression GO network. PPIs are assigned to each GO gene set to build the GO network. Expression profiles are used to calculate the co-expression level for two genes as weight of the edge for GO network. Once the co-expression GO network was built, we divided the network into several functional modules by the weighted GN algorithm and the whole modules by p-SAGE based on expression differential level to obtain the TRMs. Next, we screened the TRM to search the MM (Fisher’s exact test, P ≤ 0.05) and defined them as core modules.

The identification of the significant expression changed functional modules was conducted by integrating mRNA expression, protein-protein interaction and Gene Ontology (GO) function annotation (Figure 1). The identification process is described briefly as follows. Further details are provided in the Materials and Methods section.

Based on the GO annotation, the whole genes on the expression profile were classified into several smaller GO clusters, omitting the genes not present in GO. The total number of GO clusters in each cancer type was 802 – 1004, involving 2644 – 4428 genes in total, with an average size of 25 – 36 genes per cluster. For each GO cluster, we built a GO network that consisted of nodes representing the member genes, and the edges representing the protein interactions between the corresponding two linked genes and weighted with their co-expressed level which measured by PCC. The below analysis on the network weighted by Spearman correlation is also presented and similar results can be seen in the supplementary files (Additional file 1: Table S1; Additional file 2: Figure S2, S3).

For each GO network, the weighted Girvan and Newman (GN) algorithm [20] was applied and partitioned it into the discrete functional modules. There were totally 4177 – 6863 functional modules identified for all GO networks for each individual dataset derived from a different cancer type, with an average size of 4.6 - 5.0 genes per module, which represents approximately 12.8% - 18.6% of the GO clusters.

To search the modules that reflect an alteration between two different pathological conditions, we used the modified p-SAGE algorithm [21] to evaluate the multi-gene differential expression levels for all modules. The modules were ranked in ascending order based on their significant differential expression levels (P value); thus, the top ranked modules (TRMs) exhibited greater expression differences compared to the rest of the modules. For all 10 datasets, the top 100 TRMs (TRMs_100) were typical with the original p-SAGE P values < 0.0635, and TRMs_1000 with P values < 0.090.

### Reproducible specificity to cancer development of the TRMs

**Figure 2:**
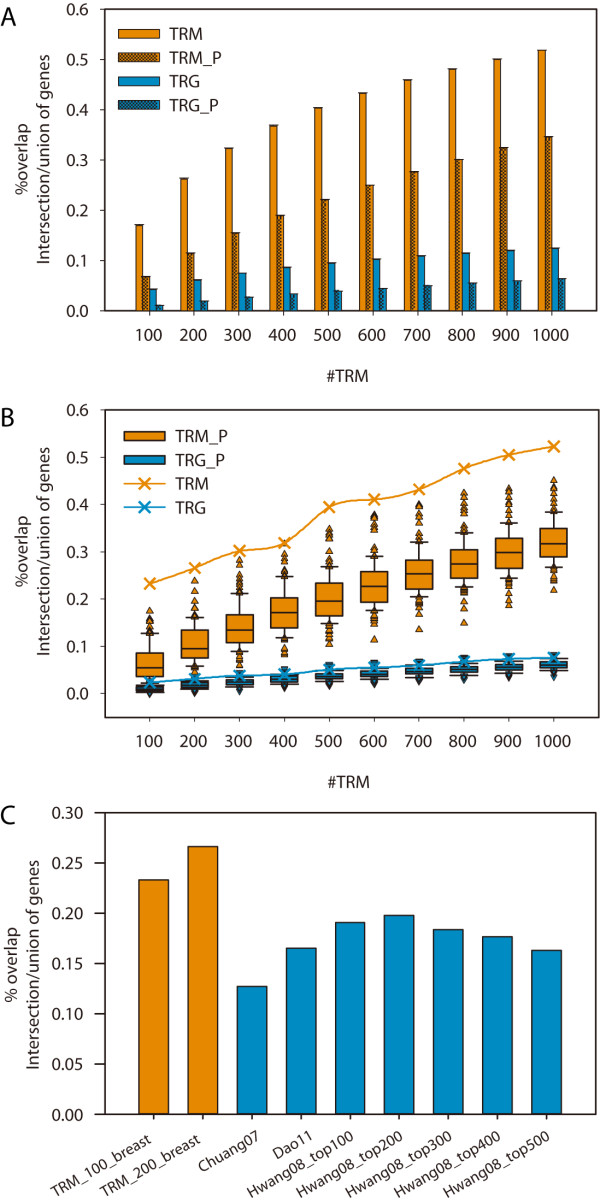
**TRM reproducibility of intra- and inter- datasets.** The percentage of overlapping genes is calculated as the ratio for the number of intersection and union of the genes. We compared the percentage of overlapping genes on TRM, TRG with the equal number of genes in TRM, and their corresponding permutation test controls (TRM_P and TRG_P). We performed the above comparison on **(A)** two randomly split halves of Lin07, **(B)** two datasets for the different microarray platform, van02 and wang05. We compared our overlapping percentage of inter-datasets (orange) with others methods (blue) **(C)**.

The intra-dataset reproducibility of the resultant TRMs was first tested using the Lin07 dataset of colon cancer samples (dataset details in Table 1). We randomly split the dataset into two halves, and estimated the robustness as the average overlapping percentage of genes in the TRMs (i.e. TRM genes) that identified from both halves across 1000 random splits. As shown in Figure 2A, the overlapping percentages for these TRMs_100-1000 (16.9% - 51.8%) were approximately four times higher than those for equal amount of individual genes that identified using *t*-test (named TRGs_100-1000, 4.3% - 12.4%). On one hand, we found that significant overlaps for 97.4% split tests of TRMs_100 and for 100% split tests of TRMs_200 – 1000, whereas approximately 10–30% split tests of TRGs_100-1000 did not exhibit a significant overlap (significance was evaluated by the Fisher’s exact test, P < =0.05). On the other hand, after permutating the sample labels ten times, i.e., a total of 10,000 split tests, we found that the overlapping percentages of TRMs with permutation (named TRM_Ps; 6.8% - 34.6%) were significantly lower compared to TRMs (Figure 2A; Wilcoxon test, P < 1E-5), and approximately 1.5-3 times higher compared to those of the TRGs (4.3% - 12.4%). Both results confirmed that the identified TRMs captured non-random changes that are specific to cancer development and distinguishable from randomness. Similar results were found in another colon cancer dataset (Barrier06) as well (Additional file 2: Figure S1).

**Table 1:** The gene expression datasets used in this study

**Name**	**Cancer type**	**#Gene**	**Platform**	**#Patients**	**Pathological settings**
Lin07	colorectal cancer	4428	Affymetrix Human Genome U133A Array	55	29 non-recurrence vs. 26 recurrence
Barrier06	colorectal cancer	4428	Affymetrix Human Genome U133A Array	50	25 non-recurrence vs. 25 recurrence
Wang05	breast cancer	4428	Affymetrix Human Genome U133A Array	286	180 non-metastasis vs. 106 metastasis
Van02	breast cancer	4203	Agilent oligonucleotide Hu25K microarray	295	217 non-metastasis vs. 78 metastasis
Jones05	clear-cell renal cell carcinoma	4428	Affymetrix Human Genome U133A Array	55	23 normal vs. 32 ccRCC
Wuttig09	clear-cell renal cell carcinoma	4428	Affymetrix Human Genome U133 Plus 2.0 Array	68	29 good prognosis vs. 39 poor prognosis
Sanchez10	non-small cell lung cancer	4428	Affymetrix Human Genome U133 Plus 2.0 Array	91	45 normal vs. 46 tumor
Beer02	non-small cell lung cancer	2644	Affymetrix Human Full Length HuGeneFL Array	86	24 dead vs. 62 alive
Riker08	melanoma	4428	Affymetrix Human Genome U133 Plus 2.0 Array	82	42 non-metastatic vs. 40 metastatic
Freije04	gliomas	4428	Affymetrix Human Genome U133A Array	85	59 dead vs. 26 alive

Next, we tested inter-dataset reproducibility using two datasets of breast cancer samples with identical pathological settings but different microarray platforms (Wang05 and Van02). We found significant overlaps of TRMs_100- 1000 from these two datasets (Fisher’s exact test, P < 5.26E-61). As shown in Figure 2B, the overlapping percentages (23.3 - 52.3%) of TRMs were significantly higher compared to those for TRGs (2.4 - 7.6%) and TRM_Ps (1.3 - 45.1%). Moreover, the overlapping percentages of TRMs were approximately two times the mean of overlapping percentages for TRM_Ps, and were significantly higher than the extreme values from the permutation tests (Figure 2B; Grubbs outlier test, p-values < 0.05), but were not found using TRGs. These results suggested a greater stability across datasets at TRMs but not the single gene level and confirmed that the TRMs were highly reproducible and specific to cancer development across two independent datasets.

Lastly, we compared the inter-dataset reproducibility with other methods. The calculations that we utilized for whole overlapping percentages from different methods were unified as the ratio between the intersection and union of the genes in the modules. As seen in Figure 2C, the overlapping percentage of TRMs (TRMs_100: 23.3%, TRMs_200: 26.6%) was much higher compared to those from other methods (Chuang07 [22]: 12.7%; Hwang08 [23]: 12.7%) with the exact same datasets and a similar number of modules, and even higher than the method (Dao11 [24]: 16.5%) for two splits of GSE20194 (more details are provided in Additional file 1: Table S2).

### Module-level enrichment of mutations in TRMs

In the following, we focused on the TRMs_100 and investigated the potential link to mutations.

**Figure 3:**
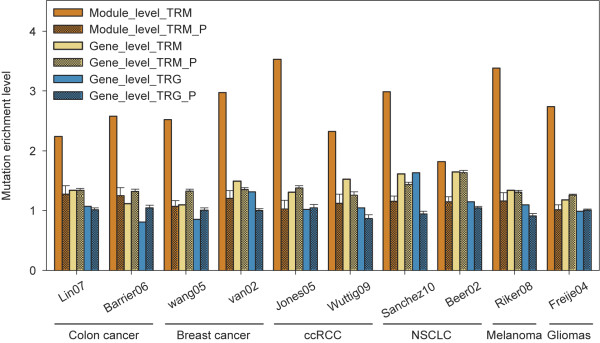
**Mutation enriched in TRM at module level.** The mutation enrichment level for TRM (Module_level_TRM) is calculated as the ratio of the number of MM in TRMs_100 and remaining modules. As a control we also performed the same analysis on the mutated genes in TRMs_100 (Gene_level_TRM), top rank *t*-test genes with the same number of genes in TRMs_100 (Gene_level_TRG), and their respective permutation test controls.

We first assessed if the TRMs_100 tended to contain a greater number of mutated genes compared to lower-ranked functional modules by calculating the ratio of their percentages of mutated genes. For all ten datasets, the ratios of TRMs_100 were slightly above one, significantly higher compared to those of TRGs (paired *t*-test; P = 1.3E-4), but not significantly different from those of TRM_Ps (paired *t*-test; P = 0.46) (Figure 3). We hypothesized that it was potentially caused by hub-genes that were preferred targets of mutations [25] and appeared more often in the identified modules; however, they were not associated with cancer development.

Alternatively, we repeated the same analyses listed above, but tested for the percentage of mutated modules (MMs) rather than mutated genes in the TRMs_100. Interestingly, we found that the mean ratio of the percentage of MMs in TRMs_100 vs. non-TRMs_100 (the ratios are referred to as MM enrichment scores hereafter) was 2.71 ± 0.53 and was significantly greater than one (P values < 7.3E-09). In contrast, the mean ratio for TRM_Ps was over two times lower (Figure 3; Module_level_TRM_Ps) and remained similar to those for mutated gene percentages in non-permutated or permutated cases (Figure 3; Gene_level_TRM and Gene_level_TRM_Ps). These findings were consistent for all of the cancer types that we examined in this study, suggesting that mutations were non-randomly linked to cancer development via TRMs, and were not necessarily dependent on the number of genes mutated in each TRM.

We evaluated the sensitivity of MM enrichment score to the changes of the pathological setting within the same cancer type. We found the much smaller difference of MM enrichment scores in colon cancer (0.34) and breast cancer (0.46), both of which had same pathological settings, although different microarray platform were utilized in breast cancer. However, approximately two fold greater differences were found in non-small cell lung cancer (NSCLC) datasets (1.17) and clear-cell renal cell carcinoma (ccRCC) datasets (1.21). In each of these two cancer types, the datasets utilized different pathological settings (details in Table 1) as well as different microarray platforms. Taken together, these findings suggested that the MM enrichment scores in TRMs_100 are reproducible and more sensitive to changes in the tumor developmental stages compared to microarray profiling methods.

### Informative marker of driver mutations: core modules

As we hypothesized, core modules, i.e. the modules with significant genomic and transcriptomic changes, may be more prone to containing driver mutations. To test this idea, we first analyzed the percentage and distribution of the known cancer drivers in these core modules. We used three cohorts of CAN-genes identified using a frequency-based approach and viewed as the most likely candidate cancer drivers in colon cancer [26], breast cancer [26], and gliomas [17]. We mapped these CAN-genes onto these cores modules identified from five datasets of these three types of cancers.

Overall, all of these three cancer types had over 50% core modules that contained at least one CAN-gene (Barrier06: 88.9%; Lin07: 50%; van02: 81.8%; and Freije04:52.4%). In contrast, the percentages were significantly lower for non-“core modules” in the TRMs_100 (Fisher’s exact test, P < 0.05): Barrier06:26%; Lin07:16%; van02: 12%; and Frejie04: 9%. Moreover, due to few number of significant mutation enrichment in the TRM_Ps_100, there were even lower percentage of core modules that contained at least one CAN-gene in the permutation test (Wilcoxon test, p < 2.2e-16): Barrier06:9.6%; Lin07:10.5%; van02:8.7%; Freije04:8.4%.

Most CAN-genes in core modules are well-known cancer drivers (the complete CAN-genes list in core modules is provided in Additional file 1: Table S4); for example, APC, SMAD4 and TP53 for colorectal cancer [27] and BRCA1 for breast cancer. Moreover, it is well documented that almost all CAN-genes in core modules from gliomas exhibit critical alterations in the three important pathway, TP53 pathway (TP53), RB1 pathway (RB1 and CDKN2A), and PI3K/PTEN pathway (PIK3R1, PTEN) [17]. These results suggest that these core modules are informative of the existence of cancer drivers. Gene ontology analysis also indicates that many of these core modules are associated with cell survival and proliferation, cell cycle, metabolism, cell death or apoptosis, or response to DNA damage (details provided in Additional file 1: Table S5), events that are critically relevant to the progression of cancer [28].

### Mutated genes in core modules are hub-genes or functionally similar

Given the above findings that the core modules were non-randomly associated with tumor development at both the expression and mutation levels, and were more critical cancer drivers and biological processes, we performed network analysis on the core modules to explore the properties of their mutated genes across six different cancer types.

**Figure 4:**
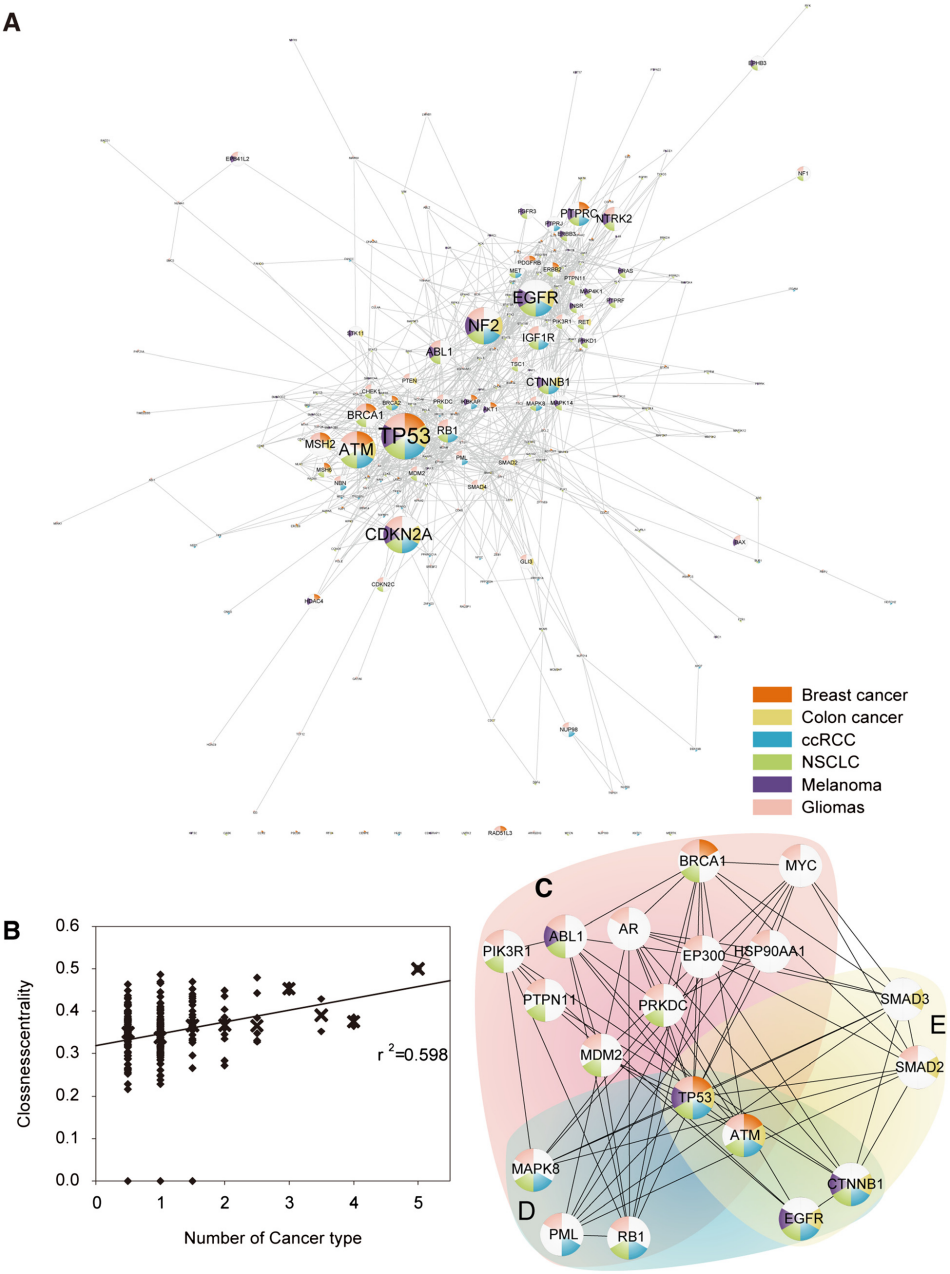
**The network properties of mutated genes in core modules.(A)** The network of the mutated genes in TRMs_100 is visualized using Cytoscape. Pie charts of each node marked their corresponding different cancer types and the size indicated the number of cancer types. **(B)** The scatter plot for the closeness vs. the number score of cancer type (diamond dot). The linear regression line was built using the medium of the closeness for each number score (x dot). The top 30 hub genes of the network (A) was mapped on gliomas **(C)**, ccRCC **(D)**, and colon cancer **(E)**, respectively.

There were a total of 236 mutated genes in the core modules identified from all ten datasets. As somatic mutations are not biased toward more genes in a TRM, one alternative possibility is that they selectively alter certain genes with pivotal roles to the module. To investigate this further, we built a network of the mutated genes in core modules with the known protein interactions (Figure 4A). We found that genes mutated for a greater number of cancer types tended to reside in more central part of the network, whereas those mutated for less cancer types were often found in the peripheral regions. Alternatively, we also found a positive linear correlation between the closeness score (definition are provided in the Materials and Methods section) of a mutated gene and the number of cancer types (Figure 4B; $R^{2}$: 0.5979, P = 0.0145). This result suggests that mutations tend to occur at hub genes with central regulatory roles.

For each cancer type, we analyzed the smaller networks formed by the mutated genes in the core modules. Interestingly, for all cancer types, the networks exhibited comparable heterogeneity, clustering coefficients, and connectivity to the original network, but approximately 2–7 times greater centralization and higher density scores (Additional file 1: Table S6). These results indicated a greater influence from the specific hub genes for each cancer type. We listed the top hub genes mutated for ccRCC, gliomas and colon cancer in Figure 4C-E, respectively. Of ccRCC, one hub gene, MAPK8, serves as a critical component of the JNK signaling pathway, which is known to be important for ccRCC. One highly conserved modulator of this pathway, SPOP, is highly expressed in 99% of ccRCC samples [29]. No comparable strong correlation was found for colon cancer or gliomas. In contrast, two hub genes of colon cancer, SMAD2 and SMAD3, are from the TGFβ/SMAD signaling pathway whose role is more established for the development of colon cancer compared to the other two cancer types. We suggested that these type-specific hub genes may reveal the specific roles of their pathways for each cancer type, as opposed to general roles in cancer development.

On the other hand, the higher density of cancer type-specific networks may imply a closer functional relationship among mutated genes in core modules from the same cancer type compared to those in different cancer types. To test this hypothesis, we generated the networks of mutated genes in core modules that identified from each of the ten cancer datasets. As expected, we found there were generally higher network relatedness scores (R, definition are provided in the Materials and Methods section) between the dataset networks from the same cancer type compared to different cancer types. These differences were significant for the shortest path cutoff = 0 (denoted as sp0 hereafter), i.e., no direct interactions, compared to cutoff = 1 or 2, presumably due to the known small world effect within biological networks. These results demonstrate that networks from the same cancer type would be functionally more closely related compared to those from different cancer types.

### Cancer type specific vs. general mutated genes in core modules

Since different network properties were identified between the mutations in core modules with higher and lower number of cancer type (N), we classified these mutations into two categories: mutated genes with N > 1 and N < =1, and further investigated them separately. Note that for cancer type with two different datasets, if a mutated gene in core modules only belonged to one of them, its number of cancer type N was defined as 1/2.

We initially investigated whether the relatedness scores could be attributed to the mutated genes (with N < =1) in core modules specific to a certain cancer type. After the removal of genes with N > 1, the remaining networks of melanoma or gliomas showed no relatedness to those of other cancer types. In contrast, lung cancer showed considerable relatedness scores (0.02-0.073) to other cancer types (except gliomas and melanoma) comparable to their within-type relatedness (0.0508). The only other cross-type non-zero relatedness was between one breast cancer dataset and one colon cancer dataset (0.053). Interestingly, the two datasets from breast cancer (0.087), colon cancer (0.12), and ccRCC (0.14) exhibited relatively higher non-zero within-type relatedness, respectively. These findings indicated that type-specific mutated genes in core modules are less functionally linked to those of other cancer types except for lung cancer.

**Figure 5:**
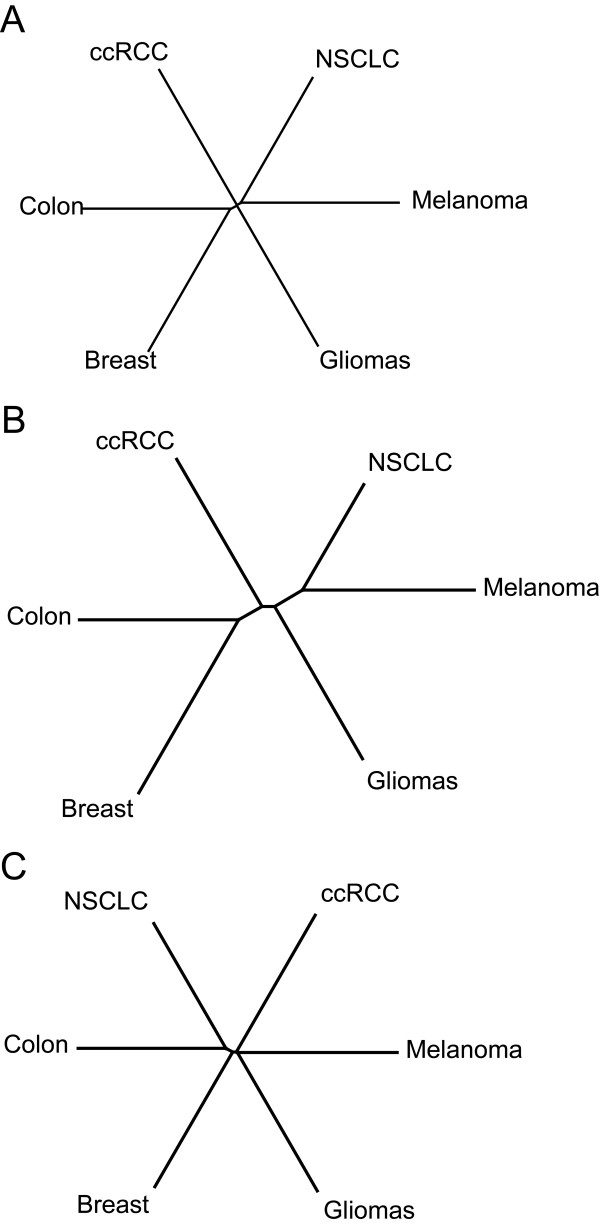
**The phylogenic relationship tree of the six cancer types.** Using the tree viewer software Dendroscope, we display the three phylogeny trees: **(A)** totTree; **(B)** comTree; **(C)** rareTree.

We next performed a systematic evaluation of the roles of the two kinds of mutated genes in core modules for different cancer types and the three Neighbor-Joining phylogenies of the six cancer types based on their network relatedness scores were derived from (further details are provided in the Materials and Methods section): the mutated genes in core modules with N > 1 (comTree), the mutated genes in core modules with N ≤ 1 (rareTree), and all 263 mutated genes in core modules (totTree) as control (Figure 5A-C). Interestingly, we found that the totTree and rareTree were almost identical to a star-like topology in which each cancer type is about equally distant from one another. In contrast, the comTree clearly showed two more closely paired cancer types. One pair contained breast cancer and colon cancer, which is consistent in that both cancers are adenocarcinoma. The other pair contained lung cancer and melanoma. Melanoma originates from neural crest cells and 10%–30% of the NSCLC cells express prominent neuroendocrine features [30]. Therefore, the comTree may reveal the shared neural features between the two cancer types. In support of this hypothesis, we also found the nearest cancer type to be gliomas, a cancer that develops in the nervous system. Taken together, these results suggest that most mutated genes in the core modules specific for each cancer type are highly functionally independent, whereas those present in multiple cancer types potentially reflect a relatedness of these cancer types, e.g., similar cells of origin.

Lastly, to obtain the two stable sets of mutated genes, type-specific and general mutated genes, we tested whether the number of cancer types for each mutated gene in the core modules was not random by permutated labels of tumors 100 times. Note that in each of permutation tests, once any of these mutated genes did fall into the core modules, its number of cancer types was defined as zero. The results indicated that almost all (about 97.9%) mutated genes in core modules passed the significance test except for five genes (Wilcoxon test; p < 0.01). Of the genes that passed the permutation test, we narrowed the genes down to 11 outlier genes with N > 1, termed general mutated genes, and 19 outlier genes with N ≤ 1, termed type-specific mutated genes (one-tail p < 0.05 from Grubbs test) (Table 2).

**Table 2:** General versus type-specific mutated genes

**Mutation type**	**Caner type**	**Gene lists**
General	Breast cancer	TP53 CDKN2A ATM RAD51L3
	Colon cancer	TP53 CDKN2A ATM RET STK11
	ccRCC	TP53 CDKN2A ATM PTPRJ
	NSCLC	TP53 CDKN2A ATM RAD51L3 NTRK2 PRKDC RET EPHB3
	melanoma	TP53 CDKN2A NTRK2 PRKDC STK11 EPB41L2 PTPRJ EPHB3
	gliomas	TP53 CDKN2A ATM RAD51L3 NTRK2 PRKDC RET EPB41L2
Type specific	Breast cancer	CDC27 CENPE DNAJA3
	Colon cancer	CDK8
	ccRCC	HUS1 FANCC
	NSCLC	HUS1 CDK8 CASK
	melanoma	IL4R PAK1 CDK5RAP1 PRC1 KIF5C KRT17
	gliomas	ID3 ARHGDIG GATA6 AR RALBP1 MTA1

Of the 11 general mutated genes, there were six tumor suppressor genes and four oncogenes, including the TP53 and CDKN2A found in core modules mutated in all cancer types. Most of these genes are related to the well-known pathways involved in cancer development. The exceptions are three genes (NTRK2, EPHB3, and EPB41L2), which are known to play a role in neural systems, and only appear in some of the core modules from the two cancer types of neural origin, gliomas and melanoma, and NSCLC that expresses prominent features of neuroendocrine cells. This finding again confirmed a closer relationship of these three cancer types in the comTree phylogeny.

Of the 19 type-specific mutated genes, CDK8 has been established as a colorectal cancer oncogene that regulates beta-catenin activity [31] and CDK5RAP1 located on the melanoma-susceptibility region [32]. The gliomas and melanoma contained at least twice as many as the other types. Particularly, the melanoma type specific mutated gene KRT17 encodes the keratin 17 which belongs to a group of fibrous proteins forming the structural framework of certain cells, particularly for the skin. The gliomas type-specific mutated gene AR encodes the androgen receptor, whose role in the development of neural systems has been well established. We found that genes in this category were studied relatively less for their roles in cancer development; however, our findings here indicate a potential functional role for them in which additional studies are warranted.

## Discussion

In our study, we introduced a novel network-based approach to discover the driver mutations during cancer development. Compared with current approaches, there are some notable features to this approach. Firstly, it has successfully allowed for the detection of critical mutations despite the frequency and for identification of the responsive core modules from the perturbed pathways or gene sets, which improves the efficiency and avoids the use of irrelevant members. Secondly, this method is based upon carefully constructed, high-quality molecular networks derived from HPRD, literature curated, and manually screened networks. In this novel network, false positive interactions are theoretically further reduced by cutting the inter-GO connections and weighting the interactions using co-expression values, as opposed to other networks which are inferred by using only the co-expression levels [9] or solely literature curated networks obtained from different contexts [10]. Additionally, our approach is based on an explicit hypothesis that phenotypic changes represented by significant transcriptomic changes respond to cancer driver mutations. Unlike other methods that integrate gene expression information only to infer the modular network structures [9], we also used the differentially expressed levels of the modules as a tool to screen the modules most likely influenced by drivers, to characterize those core modules, and to identify mutations enriched in the modules.

Our findings demonstrated a correlation between genetic mutations and phenotypic alterations at the module level, not at the single gene level. These genotype-phenotype correlations have been conceived for a long time but were only partially probed previously in certain genes, e.g., EGFR, TP53, BRCA1, BRCA2, K-ras, and their pathways. This may indicate that while the impaired DNA repair pathways seems to result in mutations widely distributed over all genes, it also causes more damage or the most responsiveness in the core modules. The presence of core modules in all six cancer types suggests the potential for a general mechanism, which supports the hypothesis that cells are modularly organized and module disruption potentially causes cancer.

Furthermore, the robustness of these findings has also been demonstrated. On one hand, we compared different strategy’s impact on the results. In details, when weighting GO network, we used two different co-expression level definitions, PCC and SCC. We compared the results using these two different strategy and found these two strategy’s results are same or similar, including network/module properties (Additional file 1: Table S1), module robustness (PCC: Figure 2; SCC: Additional file 2: Figure S1), and mutation enrichment level (PCC: Figure 3; SCC: Additional file 2: Figure S3). All results together suggested the findings are stable even though using different network weight strategy. On the other hand, we also demonstrated our findings robust by analysis ten different datasets from six cancer types, which has shown consistence.

Regarding the reason behind our findings about genotype-phenotype correlated changes, it may be attributed to co-evolution at network/module level. Proteins perform its function by interacting with other partners in the modular mode, where modularity is deemed to affect the co-evolution on the proteins [33]. First, interaction proteins have been found to be co-evolved to meet the structural constraints on the binding site [34]. Second, the member genes in the module would co-evolve to be co-opted for a new common function [35]. Thus, protein-protein interaction information, especially the modularity, contributes to build the relationship between genotype and phenotype.

Core modules provide biological insight into cancer development. Firstly, core modules are useful to identify the cancer drivers, which have been demonstrated in all three cancer types in which driver mutation data are available. Secondly, mutated genes in core modules tend to be hub-genes and functionally similar. Closer links among mutated genes were found in core modules from the same cancer type. Also, higher network relatedness was found between two different datasets from colon cancer (0.242) and ccRCC (0.256) compared to breast cancer (0.086) and NSCLC (0.096). This may imply a more complex development of breast cancer and NSCLC compared to colon cancer and ccRCC, or alternatively, it may be due to the heterogeneous histopathological features within their corresponding datasets. For breast cancer, samples in wang05 [36] were lymph node-negative whereas the combination of lymph node-negative and positive were found in van02 [37]. For NSCLC, Sanchez10 [38] contained primary adenocarcinomas and squamous-cell carcinomas whereas only primary adenocarcinomas were found in Beer02 [39]. Thirdly, for the mutated genes in the core modules from multiple cancer types, some may play a central role in cancer pathways such as TP53. Also, these genes’ network relativeness based cancer phylogenic relationship reflects the similar cellular origins across the different cancer types, which may be due to epigenetic factors, e.g. (1) common mutational mechanism pre-disposed at the early stage of differentiation for certain cell types, or (2) similar challenges from tumor microenvironment. This finding is also consistent with the prior findings that tissue lineages can influence mutational frequencies of certain oncogenes [40,41].

However, there is not sufficient evidence to make conclusions regarding the causal relationship of mutations and expression changes, and many mutated genes within the core modules may only be associative. In addition, due to the public data limitation, the tumor sample sources exhibit differences between the expression profile and genomic mutation data. Besides, the pathological conditions are different between different datasets even though the results have demonstrated that networks from same cancer types, whether or not with same or different pathological status, have higher network relatedness than those from different cancer types, suggesting the differences from cancer types dominated the comparison between different cancers. Along with the rapidly increasing amount of data available, some aspects of our approach can be augmented by incorporating data from other dimensions, e.g., copy number variations or epigenetics, which could potentially help reduce the false positive rate and identify more explicit pathways. Meanwhile, more full datasets for each patient under each pathological condition will become available in the future. The core modules revealed in this study are potentially valuable resources for the elucidation of how mutations arise, with general or specific roles in different cancer types, and provide insight into convergent cancer development in different organs, and may be informative for clinical usage as well.

## Conclusions

In summary, the correlations between genetic and phenotypic changes were successfully detected on the core modules for all ten datasets from six cancer types. Meanwhile, an effective network-based approach was proposed to identify driver mutations from core modules with convergent genotype-phenotype changes, and its utility was demonstrated. Furthermore, through comprehensive network analysis on the mutated genes in core modules from these six cancer types, we found different properties between cancer type specific and general mutated genes: the majority were cancer type specific and relatively functionally isolated from those found in other cancer types; the rest were general mutated genes and tended to be the hub genes, and can reflect the phylogenetic relationship between the corresponding cancers, providing critical insights into convergent cancer development in different organs.

## Methods

### Data source

The protein-protein interaction network data were obtained and combined from HPRD database release 8 [42] and BioGRID database [43]. All redundant edges were collapsed into single edges. The final network covered 6511 nodes and 29684 interactions.

Gene expression datasets utilized were the public pre-processed datasets that included six cancer types and 10 datasets in total: colorectal cancer (Lin07 [25], Barrier06 [44]), breast cancer (Wang05 [36], Van02 [28]), clear-cell renal cell carcinoma (ccRCC, Jones05 [45], Wuttig09 [46]), non-small cell lung cancer(NSCLC, Sanchez10 [38], Beer02 [39]), melanoma (Riker08 [47]), gliomas(Freije04 [48]) (details were provided in Table 1). Missing values for each probe were filled by applied R package ‘impute’ [49]. For genes with multiple probes, final expression values were the mean of the expression levels for multiple probes.

Gene annotations were obtained from C5 Gene Ontology (GO) sets of the Molecular Signatures Database (MSigDB) v2.5 [50]. The C5 GO gene sets are based on GO terms but removed those with too large (e.g. biological processes), too small (<10 genes), or redundant gene sets.

The mutated genes for each cancer type were downloaded from the COSMIC website (http://www.sanger.ac.uk/cosmic/) [51]. This database stored cancer mutation information which included manually curated data from the published scientific literature, and the output from the Cancer Genome Project (CGP) at the Sanger Institute for in situ tumors or cultured cancer cell lines. For more details, see Additional file 1: Table S3.

### The procedures for identifying core modules

For each dataset, core modules were identified as follows (Figure 2).

Step 1. Constructing the co-expression networks for each GO cluster

We build the networks for each GO cluster. It has been previously demonstrated that integrating the prior functional information (e.g. GO) can contribute to the identification of functional modules [50,52-54]. In this way, the multi-functional genes are not restricted as one functional module, and can eliminate the inter-GO connections while retaining interactions within GO. The retained interactions are potentially functional in vivo and reduce false positive interactions. To build the network of GO clusters, we first created the non-connected GO network with the nodes represented by whole overlapped genes of GO sets and expression profiles. Next, for each node in the network, we retrieved the protein-protein interaction network for all neighbors within the GO cluster and placed the interactions into the GO network.

Regarding the weight for each edge in the network, co-expression level measured by the Pearson Correlation Coefficient (PCC) was used to quantify the similarity between the two corresponding linked nodes and weight of each edge on the GO network, since co-expression level allowed for approximations reflective of the similarity and proximity between the two linked genes [55]. Notably, the co-expression level was specific to each individual dataset. Besides Pearson Correlation Coefficient (PCC),other metrics, such as mutual information and Spearman correlation were utilized as alternatives to calculate the gene co-expression levels because it has been previously shown that networks based on different co-expression level metrics exhibit similar network properties [56]. Moreover, PCC indicates the collaborative degree of two genes’ expression levels, rather than the individual strength, and is an appropriate tool for analysis of microarray data that usually contain considerable noise [57]. Here, the co-expression similarity *S*_*sj*_ is the absolute value of the PCC between gene expression profiles × _i,_ ×_i_

(1) $$S_{\mathrm{ij}}=\left|cor(x_{i},x_{j})\right|$$

Step 2. Module discovery using the weighted GN algorithm

We applied the widely used weighted Girvan and Newman (GN) algorithm [20], a graph theory method based on edge betweenness algorithm. The weighted GN algorithm is an edge-oriented and globally optimal-search method in large number of identification methods [22,58-62]. The idea is that the few edges lying between the modules pose ‘traffic bottlenecks’ when travelling from one module to another; thus, if these edges can be identified and removed, the networks will be naturally separated into isolated parts. In detail, the betweenness of all edges in the weighted GO network is calculated based on the sum of the all shortest paths through them and divided by the their corresponding weights. The edge with highest betweenness is removed. The betweenness of all remaining edges was recalculated and the edge with the highest betweenness was removed repeatedly until no edges remained. Considering that the original GN algorithm cut point with the highest Q value led to a huge disequilibrium of module sizes and relatively lower biological coherence in modules exhibiting a relatively larger size [63], we performed a minor adjustment by setting a size limit of less than 20 for each module and by ignoring the modules with only one isolated gene.

Step 3. Ranking the modules based on their differential expressions

We computed the differential expression levels of the identified multi-gene modules in the two pathological conditions of the dataset using the P-SAGE algorithm [21], which we previously developed. The p-SAGE method used the quadratic sum of the t score to evaluate the differentially expressed levels and obtain the significance level (P value) for each gene set, because the quadratic sum SDS follows the chi square distribution (~$\chi^{2}(n)$, where n is the size of the module) [21]. In contrast, most methods currently used [22,50,59,64] focus on averaging the expression levels or differential statistics according to the size of the module, which simply expands the statistical test of a single gene to multiple genes.

In p-SAGE, for module S with total n genes, its SDS was defined by:

(2) $$SDS(s)=\sum_{i=1}^{n}T_{i}{}^{2}$$

where *T*_*i*_ is the t score for ith gene in the module S. The significance levels (P values) of differentially expressed levels for module S were calculated using the chi-square test. Modules were then ranked based on the resultant P value in ascending order.

Step 4. Overlapping the MMs with the TRMs to obtain the “core modules”

For each module, we counted the number of mutated genes and determined whether there were significantly more mutations in the module using the Fisher’s exact test (P < =0.05). Modules exhibiting a significantly greater number of mutations were defined as mutated modules (MM). We defined the overlapped MMs with TRMs_100 as the core modules for further exploration.

### Network topological features

The mutated network analysis was performed using NetworkAnalyzer [65] and CentiScape [66], the plugins of the software Cytoscape [67].

Clustering coefficient is defined as

(3) $$C_{n}=\frac{2e_{n}}{k_{n}(k_{n}-1)}$$

where *k*_*n*_ is the number of neighbors or degree of the node n, *e*_*n*_ is the number of connections among all neighbors of the node n. The value listed in Additional file 1: Table S6 is the average of the clustering coefficient of all nodes.

Density is the normalized average number of neighbors, which indicates how dense the network is. The network with no edge has the density of 0 and the fully connected network has the density of 1.

(4) $$Density=\frac{\sum_{i}\sum_{j}a_{\mathrm{ij}}}{N(N-1)}=\frac{mean(k)}{N-1}$$

Network centralization is the index of the connectivity distribution, defined as

(5) $$Centralization=\frac{N}{N-2}(\frac{\max(k)}{N-1}-Density)$$

For example, the star network has a centralization of 1, whereas a more decentralized network has a centralization closer to 0.

Network heterogeneity is a measure of the variance of the connectivity distribution.

(6) $$Heterogeneity=\frac{\sqrt{\mathrm{var}iance(k)}}{mean(k)}$$

Closeness, a parameter utilized in Figure 4B, is defined as

(7) $$Closeness(v)=\frac{1}{\sum_{w\in V}dist(v,w)}$$

in which the denominator is the sum of the shortest path between the node v and all other nodes in the graph. A node with high closeness is usually central to the network. From a biological perspective, the closeness can be interpreted as the functional relevance of one gene upon others, and genes with higher closeness scores are more central to the regulation of other proteins.

### Cancer phylogeny tree

The distance matrix (D) for two cancer types was converted from a similarity matrix that is the network relatedness matrix (R). The value *R*_*ij*_ in the network relatedness matrix is calculated as follows:

(8) $$R_{\mathrm{ij}}=\frac{N_{0ij}}{N_{1ij}}$$

Where *N*_0*ij*_ is the count of the shortest path whose length was not beyond a cutoff (0, 1, or 2) between nodes from ith and jth dataset networks, and *N*_*1ij*_ is this count in their combined network.

The value *D*_*ij*_ in the distance matrix D is calculated as follows:

(9) $$D_{\mathrm{ij}}=1-R_{\mathrm{ij}}$$

The distance matrix was used to build the phylogeny tress for different cancer types using the PHYLIP 3.67, which is an implementation of the neighbor-joining method.

## Abbreviations

BioGrid, Biological general repository for interaction datasets; CAN genes, Candidate cancer genes; ccRCC, Clear Cell Renal Cell Carcinoma; CGP, Cancer Genome Project; COSMIC, Catalogue of Somatic Mutations in Cancer; GN, Girvan and Newman; GO, Gene ontology; HPRD, Human protein reference database; MM, Mutated module; MSigDB, Molecular Signatures Database; NSCLC, Non-small-cell lung carcinoma; PCC, Pearson Correlation Coefficient; PPI, Protein-protein interaction; p-SAGE, Parametric statistical analysis of gene sets; TRG, Top ranked gene; TRG_P, Top ranked gene after permutation; TRM, Top ranked module; TRM_P, Top ranked module after permutation.

## Competing interests

The authors declare that they have no competing interests.

## Authors’ contributions

WL conceived and designed the study, implemented the method, collected the results, drafted and revised the manuscript. RW participated in the design of the study and the display of the results. LB designed the illustration figures and assisted to revise the manuscript. ZY participated in revising the manuscript. ZS discussed on design of study and revised the manuscript. All authors read and approved the final manuscript.

## Supplementary Material

Additional file 1: **Table S1.** Lists summary of the modules identified from the network weighted by the Pearson vs Spearman correlation, respectively. **Table S2.** Lists the inter-datasets reproducibility results (overlapping percentage) from different methods.**Table S3.** Lists the detailed options about the mutation data from the COSMIC. **Table S4.**lists the CAN-genes in core modules. **Table S5.** lists the GO summary of the core modules in all cancer types. **Table S6.** lists the network features of the mutated genes in the core modules. Click here for file

Additional file 2: **Figure S1.** Shows the intra-dataset reproducibility results of TRMs identified from the Barrier datasets. **Figure S2.** Shows inter-dataset reproducibility results of TRMs identified from two breast cancer datasets (edge weighted by the spearman correlation). **Figure S3.** Shows the TRMs’ mutation enrichment at module level (edge weighted by the spearman correlation). Click here for file
