# Supplementary material for: Cancer core modules identification through genomic and transcriptomic changes correlation detection at network level
Source: BMC Syst Biol. 2012 Jun 12;6:64. doi: 10.1186/1752-0509-6-64 (PMC3443057; doi:10.1186/1752-0509-6-64)

Additional File 1

Supplemental Figure S1. TRMs Reproducibility (TRMs identified using Barrier06 datasets). We compared the percentage of overlapping genes on TRM, conventional t test analysis (TRG) with the same number of genes in TRM, and their corresponding permutation test controls (TRM_P and TRG_P)

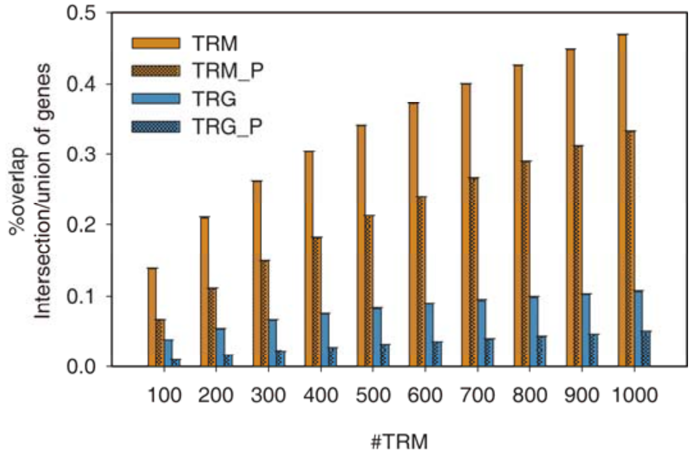

Supplemental Figure S2. TRMs Reproducibility across two breast cancer datasets, van02 and wang05 (Edge weighted by the spearman correlation). The percentage of overlapping genes is calculated as the ratio for the number of intersection and union of the genes. We compared the percentage of overlapping genes on TRM, conventional t test analysis (TRG) with the same number of genes in TRM, and their corresponding permutation test controls (TRM_P and TRG_P).

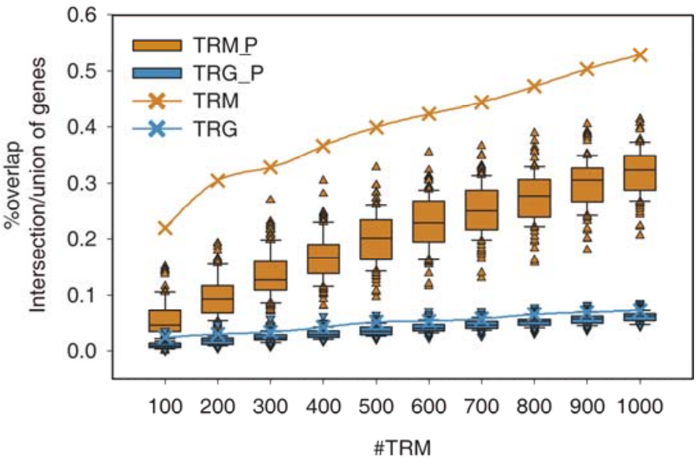

Supplemental Figure S3. Mutation enriched in TRM at module level (Edge weighted by the spearman correlation). The mutation enrichment level for TRM (module_level_TRM) is calculated as the ratio of the number of MM in TRMs_100 and remaining modules. As control we also do the same analysis on the mutated genes in TRMs_100 (gene_level_TRM), top rank t-test genes with the same number of genes in TRMs_100 (gene_level_TRG), and their respective permutation test controls.

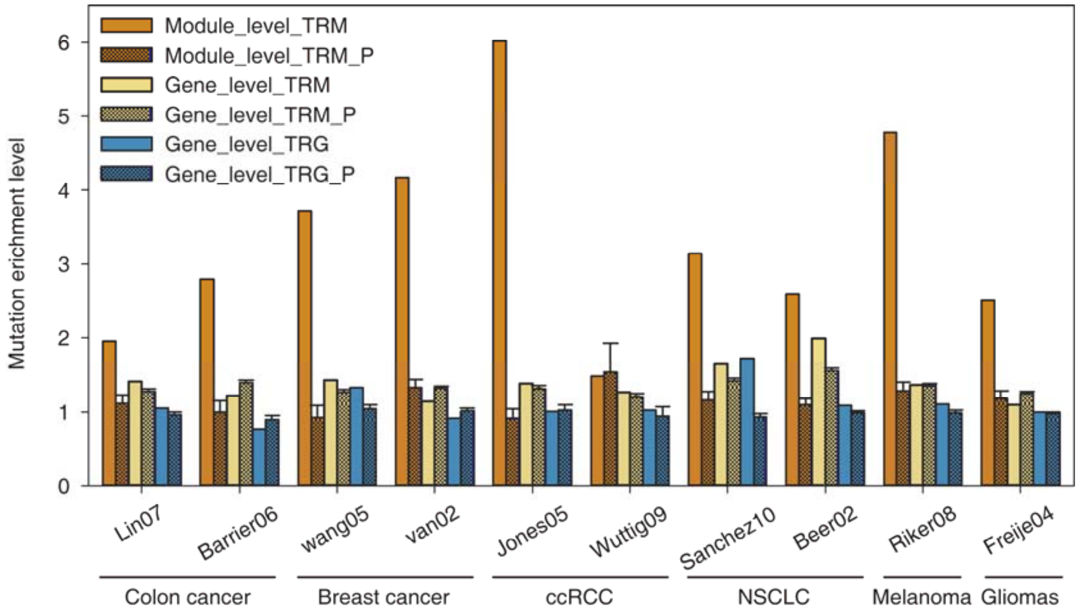

Supplement: Additional file 1: — Table S1. Lists summary of the modules identified from the network weighted by the Pearson vs Spearman correlation, respectively. Table S2. Lists the inter-datasets reproducibility results (overlapping percentage) from different methods.Table S3. Lists the detailed options about the mutation data from the COSMIC. Table S4.lists the CAN-genes in core modules. Table S5. lists the GO summary of the core modules in all cancer types. Table S6. lists the network features of the mutated genes in the core modules. [file 1752-0509-6-64-S1.pdf]
