# Supplementary material for: Cancer core modules identification through genomic and transcriptomic changes correlation detection at network level
Source: BMC Syst Biol. 2012 Jun 12;6:64. doi: 10.1186/1752-0509-6-64 (PMC3443057; doi:10.1186/1752-0509-6-64)
Supplement: Additional file 2: — Figure S1. Shows the intra-dataset reproducibility results of TRMs identified from the Barrier datasets. Figure S2. Shows inter-dataset reproducibility results of TRMs identified from two breast cancer datasets (edge weighted by the spearman correlation). Figure S3. Shows the TRMs’ mutation enrichment at module level (edge weighted by the spearman correlation). [file 1752-0509-6-64-S2.pdf]

Additional File 2

Supplemental Table S1. Summary of the modules and comparison between the Pearson vs Spearman correlation

name	cancer type	#GO_networks	#genes_in_GO_networks	average_per_GO	#modules		#genes		average_#genes_per_module		p_value_of_TRMs_100		p_value_of_TRMs_1000		%GO_networks	
					Pearson	Spearman	Pearson	Spearman	Pearson	Spearman	Pearson	Spearman	Pearson	Spearman	Pearson	Spearman
Lin07	colorectal cancer	993	4428	34.32	6742	6721	3121	3122	4.83	4.84	3.49E-09	2.75E-09	5.55E-04	5.89E-04	14.09%	14.11%
Barrier06	colorectal cancer	993	4428	35.32	6724	6639	3134	3143	4.82	4.93	2.69E-06	1.28E-06	1.70E-03	1.60E-03	13.66%	13.96%
wang05	breast cancer	993	4428	36.32	6748	6761	3133	3124	4.81	4.79	9.67E-06	8.11E-06	1.14E-02	1.18E-02	13.25%	13.18%
van02	breast cancer	987	4203	32.95	6515	6426	2976	2980	4.74	4.83	1.22E-10	5.54E-11	6.50E-04	6.50E-04	14.39%	14.66%
Jones05	ccRCC	993	4428	36.32	6663	6690	3158	3157	4.95	4.94	0.00E+00	0.00E+00	4.09E-165	4.09E-165	13.63%	13.59%
Wuttig09	ccRCC	1004	4428	34.03	6643	6584	3180	3198	5.08	5.14	6.35E-03	6.72E-03	8.30E-02	8.20E-02	14.94%	15.11%
Sanchez10	NSCLC	1004	4428	34.03	6750	6773	3122	3119	4.80	4.78	2.09E-300	1.96E-306	1.32E-82	4.72E-82	14.09%	14.05%
Beer02	NSCLC	812	2644	24.98	4177	4166	1891	1877	4.65	4.65	2.07E-03	1.94E-03	8.98E-02	9.09E-02	18.61%	18.61%
Riker08	melanoma	1004	4428	34.03	6745	6792	3144	3145	4.85	4.83	2.75E-62	4.45E-63	1.06E-19	8.63E-20	14.25%	14.20%
Freije04	gliomas	993	4428	36.32	6863	6857	3095	3127	4.66	4.75	1.79E-06	1.21E-06	3.19E-03	3.10E-03	12.83%	13.09%

Supplemental Table S2. Comparison on overlapping percentage from inter-datasets

	overlap_percentage	#overlap	#dataset1	#dataset2
TRM_100_b	0.232747	172	443	468
TRM_200_b	0.26594	317	749	760
Chuang07	0.127219	172	618	906
Dao11	0.165254	39	145	130
Hwang08_top100	0.190476	32	100	100
Hwang08_top200	0.197605	66	200	200
Hwang08_top300	0.183432	93	300	300
Hwang08_top400	0.176471	120	400	400
Hwang08_top500	0.162791	140	500	500

Supplemental Table S3. Detailed options about the mutation data from the COSMIC

name	cancer type	tissue	subtissue	histology	subhistology
Lin07	colorectal cancer	large intestine	Except anus and appendix	adenoma and carcinoma	NA
Barrier06	colorectal cancer	large intestine	Except anus and appendix	adenoma and carcinoma	NA
Wang05	breast cancer	breast	NA	carcinoma	NA
Van02	breast cancer	breast	NA	carcinoma	NA
Jones05	clear-cell renal cell carcinoma	kidney	NA	carcinoma	clear cell renal cell carcinoma
Wuttig09	clear-cell renal cell carcinoma	kidney	NA	carcinoma	clear cell renal cell carcinoma
Sanchez10	non-small cell lung cancer	lung	NA	carcinoma	adenocarcinoma+squamous cell carcinoma+non small cell carcinoma
Beer02	non-small cell lung cancer	lung	NA	carcinoma	adenocarcinoma+non small cell carcinoma
Riker08	melonoma	skin	NA	malignant melanoma	NA
Freije04	gliomas	central nervous system	NA	glioma	NA

Supplemental Table S4. CAN-genes in core modules

cancer type	datasets	CAN_gene
colorectal	Barrier06	APC SMAD4 TP53
	Lin07	RET PTEN ERCC6 TP53 MAP2K7 MCM3 AP
breast	wang05	NOTCH1
	van02	MRE11A TIMELESS HDAC4 BRCA1
iomas	Freije04	PTEN CDKN2A RB1 PIK3R1 TP53 NF1

Supplemental Table S5. GO summary of the core modules in all cancer types.

cancer type	datasets	#driver_modules	#GO_terms	GO_terms
colorectal cancer	Lin07	8	7	REGULATION_OF_CELL_CYCLE
				PROTEIN_COMPLEX
				DNA_REPAIR
				RESPONSE_TO_DNA_DAMAGE_STIMULUS
				MACROMOLECULAR_COMPLEX
				DNA_METABOLIC_PROCESS
				SEQUENCE_SPECIFIC_DNA_BINDING
	Barrier06	9	9	STRESS_ACTIVATED_PROTEIN_KINASE_SIGNALING_PATHWAY
				JNK_CASCADE
				PROTEIN_COMPLEX
				REGULATION_OF_MOLECULAR_FUNCTION
				MULTICELLULAR_ORGANISMAL_DEVELOPMENT
				NUCLEAR_TRANSPORT
				NUCLEOCYTOPLASMIC_TRANSPORT
				CELL_CYCLE_GO_0007049
				NEGATIVE_REGULATION_OF_CELL_CYCLE
breast cancer	wang05	12	11	MITOSIS
				M_PHASE_OF_MITOTIC_CELL_CYCLE
				RESPONSE_TO_DNA_DAMAGE_STIMULUS
				DNA_REPAIR
				DNA_METABOLIC_PROCESS
				RESPONSE_TO_ENDOGENOUS_STIMULUS
				PROTEIN_COMPLEX
				ORGANELLE_PART
				INTRACELLULAR_ORGANELLE_PART
				RESPONSE_TO_STRESS
				DNA_RECOMBINATION
	van02	11	11	MITOSIS
				M_PHASE_OF_MITOTIC_CELL_CYCLE
				PROGRAMMED_CELL_DEATH
				APOPTOSIS_GO
				POSITIVE_REGULATION_OF_BIOLOGICAL_PROCESS
				SIGNAL_TRANSDUCTION
				REGULATION_OF_DEVELOPMENTAL_PROCESS
				IDENTICAL_PROTEIN_BINDING
				POST_TRANSLATIONAL_PROTEIN_MODIFICATION
				BIOPOLYMER_MODIFICATION
				PROTEIN_MODIFICATION_PROCESS

ccRCC	Jones05	17	17	ORGANELLE_PART
				INTRINSIC_TO_PLASMA_MEMBRANE
				NON_MEMBRANE_BOUND_ORGANELLE
				NUCLEUS
				CYTOSKELETON
				INTEGRAL_TO_PLASMA_MEMBRANE
				NUCLEAR_ENVELOPE
				NUCLEOPLASM_PART
				INTRACELLULAR_NON_MEMBRANE_BOUND_ORGANELLE
				NUCLEAR_MEMBRANE
				INTRACELLULAR_ORGANELLE_PART
				PROTEIN_COMPLEX
				DNA_REPAIR
				POSITIVE_REGULATION_OF_CELL_PROLIFERATION
				BIOPOLYMER_METABOLIC_PROCESS
				SIGNAL_TRANSDUCTION
				NUCLEOBASE__NUCLEOSIDE__NUCLEOTIDE_AND_NUCLEIC_ACID_METABOLIC_PROCESS
	Wuttig09	12	11	ORGAN_DEVELOPMENT
				NUCLEUS
				RECEPTOR_ACTIVITY
				DNA_BINDING
				CYTOPLASMIC_PART
				PROTEIN_METABOLIC_PROCESS
				REGULATION_OF_BIOLOGICAL_QUALITY
				TRANSCRIPTION_FROM_RNA_POLYMERASE_II_PROMOTER
				TRANSCRIPTION__DNA_DEPENDENT
				RNA_BIOSYNTHETIC_PROCESS
				POST_TRANSLATIONAL_PROTEIN_MODIFICATION
NSCLC	Sanchez-Palencia10	32	29	ORGANELLE_PART
				INTRINSIC_TO_MEMBRANE
				NUCLEAR_PART
				NON_MEMBRANE_BOUND_ORGANELLE
				CYTOSKELETAL_PART
				NUCLEUS
				CYTOSKELETON
				INTEGRAL_TO_MEMBRANE
				INTRACELLULAR_NON_MEMBRANE_BOUND_ORGANELLE
				INTRACELLULAR_ORGANELLE_PART

				MACROMOLECULAR_COMPLEX
				PROTEIN_COMPLEX
				PLASMA_MEMBRANE_PART
				DNA_METABOLIC_PROCESS
				DNA_REPAIR
				BIOPOLYMER_METABOLIC_PROCESS
				NUCLEOBASE__NUCLEOSIDE__NUCLEOTIDE_AND_NUCLEIC_ACID_METABOLIC_PROCESS
				RESPONSE_TO_ENDOGENOUS_STIMULUS
				CELL_CYCLE_PROCESS
				CELL_CYCLE_PHASE
				RESPONSE_TO_STRESS
				CELLULAR_PROTEIN_METABOLIC_PROCESS
				PROTEIN_METABOLIC_PROCESS
				CELLULAR_MACROMOLECULE_METABOLIC_PROCESS
				RESPONSE_TO_DNA_DAMAGE_STIMULUS
				REGULATION_OF_CELL_CYCLE
				SIGNAL_TRANSDUCTION
				KINASE_ACTIVITY
				PLASMA_MEMBRANE
	Beer02	22	18	PLASMA_MEMBRANE_PART
				REGULATION_OF_MULTICELLULAR_ORGANISMAL_PROCESS
				SIGNAL_TRANSDUCTION
				CELL_SURFACE_RECEPTOR_LINKED_SIGNAL_TRANSDUCTION_GO_0007166
				SYSTEM_DEVELOPMENT
				ORGAN_DEVELOPMENT
				ANATOMICAL_STRUCTURE_DEVELOPMENT
				HYDROLASE_ACTIVITY__ACTING_ON_ESTER_BONDS
				PROTEIN_KINASE_CASCADE
				PHOSPHOPROTEIN_PHOSPHATASE_ACTIVITY
				RNA_METABOLIC_PROCESS
				BIOPOLYMER_METABOLIC_PROCESS
				ENZYME_BINDING
				INTRACELLULAR_SIGNALING_CASCADE
				HEMOPOIESIS
				IMMUNE_SYSTEM_DEVELOPMENT
				HEMOPOIETIC_OR_LYMPHOID_ORGAN_DEVELOPMENT
				PROTEIN_METABOLIC_PROCESS

melonoma	Riker08	16	15	ORGANELLE_PART
				INTRACELLULAR_ORGANELLE_PART
				ENZYME_LINKED_RECEPTOR_PROTEIN_SIGNALING_PATHWAY
				TRANSFERASE_ACTIVITY__TRANSFERRING_PHOSPHORUS_CONTAINING_GROUPS
				TRANSMEMBRANE_RECEPTOR_PROTEIN_TYROSINE_KINASE_SIGNALING_PATHWAY
				PHOSPHORYLATION
				PLASMA_MEMBRANE
				PLASMA_MEMBRANE_PART
				ORGANELLE_ORGANIZATION_AND_BIOGENESIS
				CYTOPLASM
				POSITIVE_REGULATION_OF_CATALYTIC_ACTIVITY
				REGULATION_OF_CATALYTIC_ACTIVITY
				REGULATION_OF_MOLECULAR_FUNCTION
				BIOPOLYMER_METABOLIC_PROCESS
				PROTEIN_KINASE_ACTIVITY
gliomas	Freije04	21	21	MITOTIC_CELL_CYCLE
				BIOPOLYMER_MODIFICATION
				ORGANELLE_ORGANIZATION_AND_BIOGENESIS
				ANATOMICAL_STRUCTURE_DEVELOPMENT
				NUCLEAR_TRANSPORT
				NUCLEOCYTOPLASMIC_TRANSPORT
				ENZYME_REGULATOR_ACTIVITY
				BIOPOLYMER_METABOLIC_PROCESS
				CELL_CYCLE_GO_0007049
				NEGATIVE_REGULATION_OF_CELLULAR_PROCESS
				TRANSPORT
				MACROMOLECULAR_COMPLEX_ASSEMBLY
				CELLULAR_COMPONENT_ASSEMBLY
				MULTICELLULAR_ORGANISMAL_DEVELOPMENT
				CYTOSKELETAL_PART
				PROTEIN_MODIFICATION_PROCESS
				REGULATION_OF_BIOLOGICAL_QUALITY
				SIGNAL_TRANSDUCTION
				NUCLEAR_LUMEN
				NEGATIVE_REGULATION_OF_BIOLOGICAL_PROCESS
				REGULATION_OF_CELL_GROWTH

Supplemental Table S6. The network features of the mutated genes in the core modules.

	all	breast	colon	ccRCC	NSCLC	Melanoma	gliomas
#nodes	236	39	34	43	98	44	75
#isolated nodes	15	4	1	2	4	2	3
clustering coefficient	0.311	0.302	0.393	0.245	0.356	0.293	0.281
network centralization	0.196	1.197	1.379	1.13	0.452	1.101	0.607
avg.number of neighbours	8.364	10.846	11.176	8.744	11.061	8.818	10.293
network density	0.036	0.285	0.339	0.208	0.114	0.205	0.139
network heterogeneity	1.068	1.027	1.011	1.177	0.935	1.151	1.086
